# Cerebral Cortex Hyperthyroidism of Newborn Mct8-Deficient Mice Transiently Suppressed by Lat2 Inactivation

**DOI:** 10.1371/journal.pone.0096915

**Published:** 2014-05-12

**Authors:** Bárbara Núñez, Raquel Martínez de Mena, Maria Jesus Obregon, Mariona Font-Llitjós, Virginia Nunes, Manuel Palacín, Alexandra M. Dumitrescu, Beatriz Morte, Juan Bernal

**Affiliations:** 1 Instituto de Investigaciones Biomédicas, Consejo Superior de Investigaciones Científicas (CSIC) and Universidad Autónoma de Madrid (UAM), Madrid, Spain; 2 Laboratorio de Genètica Molecular, IDIBELL, Barcelona, Spain; 3 Center for Biomedical Research on Rare Diseases (CIBERER) Unit 730, Barcelona, Spain; 4 Sección de Genética, Departamento de Ciencias Fisiológicas II, Facultad de Medicina, Universidad de Barcelona, Barcelona, Spain; 5 Institute for Research in Biomedicine (IRB), Barcelona, Spain; 6 CIBERER Unit 731, Barcelona, Spain; 7 Department of Medicine, The University of Chicago, Chicago, Illinois, United States of America; 8 CIBERER Unit 708, Madrid, Spain; University Claude Bernard Lyon 1, France

## Abstract

Thyroid hormone entry into cells is facilitated by transmembrane transporters. Mutations of the specific thyroid hormone transporter, *MCT8* (*Monocarboxylate Transporter 8, SLC16A2*) cause an X-linked syndrome of profound neurological impairment and altered thyroid function known as the Allan-Herndon-Dudley syndrome. MCT8 deficiency presumably results in failure of thyroid hormone to reach the neural target cells in adequate amounts to sustain normal brain development. However during the perinatal period the absence of Mct8 in mice induces a state of cerebral cortex hyperthyroidism, indicating increased brain access and/or retention of thyroid hormone. The contribution of other transporters to thyroid hormone metabolism and action, especially in the context of MCT8 deficiency is not clear. We have analyzed the role of the heterodimeric aminoacid transporter Lat2 (Slc7a8), in the presence or absence of Mct8, on thyroid hormone concentrations and on expression of thyroid hormone-dependent cerebral cortex genes. To this end we generated *Lat2^-/-^*, and *Mct8^-/y^Lat2* ^-/-^ mice, to compare with wild type and *Mct8^-/y^* mice during postnatal development. As described previously the single *Mct8* KO neonates had a transient increase of 3,5,3′-triiodothyronine concentration and expression of thyroid hormone target genes in the cerebral cortex. Strikingly the absence of Lat2 in the double *Mct8Lat2* KO prevented the effect of *Mct8* inactivation in newborns. The Lat2 effect was not observed from postnatal day 5 onwards. On postnatal day 21 the *Mct8* KO displayed the typical pattern of thyroid hormone concentrations in plasma, decreased cortex 3,5,3′-triiodothyronine concentration and *Hr* expression, and concomitant Lat2 inactivation produced little to no modifications. As Lat2 is expressed in neurons and in the choroid plexus, the results support a role for Lat2 in the supply of thyroid hormone to the cerebral cortex during early postnatal development.

## Introduction

Thyroid hormones (thyroxine, T4 and 3,5,3′-triiodo-L-thyronine, T3) transport through the cellular plasma membrane is facilitated by several classes of transmembrane proteins. These include the monocarboxylate transporters (MCT), the organic anion transporter polypeptides (OATP), the heterodimeric aminoacid transporters, the Na+/taurocholate cotransporting polypeptide (NTCP) and other classes of transporters [1]. MCT8 (SLC16A2) is specific for iodothyronine transport [2], [3]. *MCT8* gene mutations cause an X-linked thyroid hormone cell transport defect, also known as Allan-Herndon-Dudley syndrome, characterized by global developmental delays, profound neurological impairment, severe intellectual deficit, and altered secretion, distribution and metabolism of thyroid hormones [4]–[6]. Patients also present elevated serum T3, reduced T4 and rT3, and unaltered or slightly elevated serum TSH.

Thyroid hormone acts on the brain from early brain development [7]–[9]. The neurological impairment of MCT8 transport defect is likely due to the failure of thyroid hormone to reach the neural target cells in adequate amounts to sustain normal brain development. The syndrome is partially replicated in mice with inactivated *Mct8* gene [10], [11]. These Mct8-deficient mice display the same alterations of thyroid hormone concentrations as the patients, indicating that the absent or defective function of the Mct8 protein in mice leads to similar alterations of thyroid hormone as those found in patients. However these mice present minimal, if any, behavioral deficits of uncertain etiology [12], and do not show the anatomical alterations typical of brain hypothyroidism during postnatal development [12], [13]. Furthermore, the expression of most thyroid hormone-dependent genes in the cerebral cortex of juvenile Mct8-deficient mice is similar to the wild type (*Wt*) mice [14]. The major role of Mct8 seems to be that of facilitating the transport of T4 and T3 through the blood-brain barrier [13], [15]. However, T4 also enters the brain through another transporter from the Oatp family, Oatp1c1 (Slco1c1, or Oatp14) [16], [17]. It has been proposed that this transport route delivers T4 to the astrocytes, where T4 to T3 conversion takes place, in a reaction catalyzed by type 2 deiodinase [18]–[21]. Therefore, in the presence of functionally defective Mct8 protein, entry of circulating T3 into the brain is restricted. Nevertheless enough T3 is still formed locally in the brain to sustain thyroid hormone-dependent gene expression [14].

The *in vivo* role of other classes of thyroid hormone transporters in the brain is uncertain. As can be concluded from the paragraph above, T3 formed in the astrocytes can readily reach the neurons in the absence of Mct8, indicating that other transporters may be involved in this process. Among others, the L-type amino acid transporters 1 and 2 have been suggested to compensate for the lack of Mct8 in mice [12], and have been implicated in T3 transport in astrocytes and neurons [22]. *Lat2* is highly expressed in the postnatal mouse cerebral cortex and has been proposed to participate together with Mct8 in T3 transport in primary astrocyte culture [22]. We have generated mice deficient in both Mct8 and Lat2 to investigate whether the combined absence of both transporters impairs T3 action in the brain more severely than in the case of Mct8 deficiency alone.

Our results indicate that Lat2 is mostly dispensable for T3 action. However it was previously reported that newborn Mct8-deficient mice unexpectedly showed increased expression of thyroid hormone-dependent genes in the cerebral cortex, indicating a perinatal state of cerebral hyperthyroidism [23]. In the present work we show that *Lat2* inactivation transiently blocks this early effect of Mct8 deficiency. Furthermore, *Lat2* is expressed predominantly in neurons. The results support that Lat2 has a role in T3 delivery to neurons during the perinatal period.

## Materials and Methods

Ethics statement: All experimental procedures involving animals were performed following the European Union Council guidelines (directive 2010/63/UE) and Spanish regulations (R.D.1201/2005, and Law 32/2007) and in accordance with University of Chicago Institutional Animal Care and Use Committee. They were approved by the ethics committee of our institution (Consejo Superior de Investigaciones Científias, CSIC; approval number SAF2011-25608).

Animals were housed in temperature (22±2°C) and light (12:12 light-dark cycle; lights on at 7 a.m.) controlled conditions and had free access to food and water. Euthanasia was performed by decapitation. For *in situ* hybridization, the mice were first anesthetized with ketamine (50 µg/g BW) and medetomidine hydrochloride (0.1 µg/g BW) and perfused with 4% paraformaldehyde in 0.1 M phosphate buffered saline pH 7.4. *Mct8* (*Slc16a2*) KO mice (male genotype, *Mct8^-/y^*) originated from the line generated by one of the coauthors (AMD) [10]. Experiments were carried out on *Wt* and KO male littermates derived from back crossing of heterozygous females with *Wt* males of the C57BL/6J strain. The *Mct8* genotype was confirmed as described [13].

The *Lat2* (*Slc7a8*) KO mouse line was generated in a mixed genetic background of C57/129Ola. Briefly, a vector with homology arms of 6.1 kb and 2.3 kb was generated, and replaced part of the promoter and exon 1 of *Slc7a8* with the neomycin resistance gene. Homologous recombination was performed by GenOway (Lyon, France). Eight male chimeras were crossed with C57BL/6J females to obtain the F1 generation. F1 heterozygotes (*Slc7a 8^+^* ^/−^) were intercrossed obtaining the three possible genotypes following a mendelian frequency. A detailed description of the procedure and the full characterization of the phenotype will be reported [24]. To generate the animals used in the experiments we started by crossing *Mct8^x^* ^/*y*^ *Lat2* ^-/-^ males with *Mct8* ^-/*x*^ *Lat2* ^+/+^ females. Then *Mct8^x/y^Lat2^−/+^* males were crossed with *Mct8^-/x^Lat2^−/+^* females. From the genotypes obtained we crossed *Mct8^x/y^Lat2^+/+^* (*Wt*) males with *Mct8^-/x^Lat2^+/+^* females to generate *Wt* and *Mct8* KO male littermates. Similarly, we crossed *Mct8^x/y^Lat2^-/-^* males with *Mct8^-/x^Lat2^-/-^* females to generate *Lat2* KO and *Mct8Lat2* KO male littermates. The *Lat2* genotype was confirmed by PCR of tail DNA (36 cycles at 62 C annealing temperature) using the following primers:

forward common: 5′GGAGCGATCTGCGGAGTGA3′;

reverse Wt-specific: 5′ACAGAGTGCGCTCCTACCCT3′;

reverse KO-specific: 5′CGGTGGGCTCTATGGGTCTA3′.

This procedure generates a 457 bp fragment from the *Wt* allele and a 180 bp fragment from the null allele.

Experiments were performed on *Wt*, *Mct8* KO (*M8*), *Lat2* KO (*L2*), and *Mct8Lat2* KO (*M8L2*) at postnatal (P) day 0, P5, P15, and P21. Most experiments were performed using 8 animals per group. Trunk blood was collected in heparinized tubes after decapitation, and the liver was frozen on dry ice and stored at −80°C. The brain was removed and the neocortex was rapidly dissected out from underlying structures, divided in two halves through the sagittal cut, blotted on filter paper, weighed and frozen on dry ice. Thyroid hormone concentration in plasma and cerebral cortex was determined as previously described [25]. We used 30 µl of pooled plasma from 2–3 P0 mice, or hemi cortices pooled from 3 mice. For P5 and P21 mice we used individual 50 µl aliquots of plasma, or individual hemi cortices. Under the conditions of the assays, the limits of detection at P0 were: 1.67 ng/ml for plasma T4, 0.03 ng/ml for plasma T3, and 0.37 ng/g for cortex T3. RNA was isolated from individual hemi cortices for P5, P15, and P21 mice and from the whole cortex from individual P0 mice, and liver. Expression of the following thyroid hormone-dependent genes was measured by qPCR: *Hr* (Hairless); *Sema7a* (Semaphorin 7a); *Klf9* (Kruppel-like factor 9, also known as Basic Transcription Element Binding protein, BTEB); *Aldh1a1*, *Aldh1a3* (Aldehyde dehydrogenases 1a1 and 1a3); *Slc1a3* (glial high affinity glutamate transporter), and *Dio1* (Type 1 iodothyronine deiodinase). Procedures for RNA isolation and qPCR were identical to those previously described [14]. Data were expressed relative to the values obtained on tissues from the *Wt*, mice which were given a mean value of 1.0 after correction for 18S RNA. *In situ* mRNA hybridization analysis for *Lat2* and the double *in situ* hybridization and immunohistochemistry were performed using methods previously described in detail [26]. The ^35^S *Lat2* riboprobe was synthesized from a 390 base pairs DNA template obtained by PCR amplification with the following primers: forward 5′-GCCTGCTGTTTCCCATTATC-3′, reverse 5′-CAGGAATACAGGGCAGAAAG-3′. The antibodies used for immunohistochemistry were against the neuronal nuclear protein NeuN (Chemicon, Millipore, final dilution 1/500), and the astrocytic glial fibrillary acidic protein (GFAP, Sigma Chemical Co, final dilution 1/2000).

Data were analyzed by one-way ANOVA and the Tukey posthoc test using the GraphPad software (www.graphpad.com). The P0 data were checked for a maternal effect using a General Lineal Model with the SPSS package. This was due to the impossibility to obtain littermates of all genotypes for the experiments. As described above, the *Wt* and *Mct8* KO mice were littermates from *Mct8* ^-/x^ *Lat2* ^+/+^ mothers. The *Lat2* KO and *Mct8Lat2* KO mice were littermates from *Mct8* ^-/x^ *Lat2* ^-/-^ mothers. The possibility that any change observed, especially at P0, was due to differences in the maternal genotype was discarded by the General Lineal Model analysis.

## Results

### *Lat2* expression in the brain

**Figure 1:**
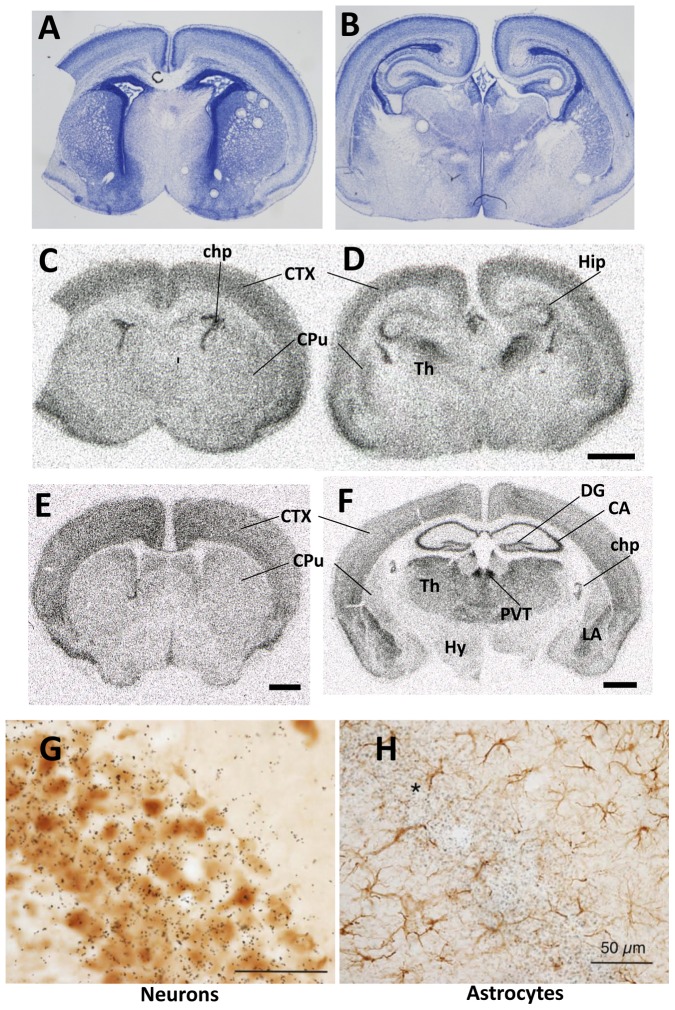
*Lat2* expression. A–D: P0: A and B are Nissl staining and C and D *in situ* hybridization radioautographs. E and F: P21 *in situ* hybridization radioautographs. Abbreviations: chp, choroid plexus; CTX, cerebral neocortex; CA, cornus Ammonis; CPu, caudate-putamen; DG, dentate gyrus; Hy, hypothalamus, Hip, hippocampus; Th, thalamus; PVT, thalamic paraventricular nucleus; LA, lateral amygdala. G: *in situ* hybridization (P21) with ^35^S-Lat2 probe combined with immunohistochemistry for NeuN to reveal neurons. Hippocampal CA1 field. H: Similar field as G, but at lower magnification, with cells stained for glial fibrillary acidic protein (GFAP). The majority of the silver grains can be seen on the neuronal pyramidal layer (asterisk) with background signal on the astrocytes. Scale bars were 1 mm in C–D, E and F, and 50 µm in G and H.

*Lat2* expression was studied in the cerebrum of P0 and P21 mice by *in situ* hybridization using a *Lat2* specific riboprobe (Fig 1). At P0 *Lat2* was expressed in neocortical layers, the piriform cortex, the hippocampus and the thalamus (Fig 1C, D). At P21, *Lat2* was expressed in the neocortex, the pririform cortex, the pyramidal and granular cell layers of the hippocampus, the amygdala, and the thalamus, with high expression in the thalamic paraventricular nucleus (Fig 1E, F). Hybridization signal was also observed in the choroid plexus of the lateral ventricles at both ages. There was little expression in the striatum and the hypothalamus. The *in situ* hybridization pattern was compatible with a predominant expression in neurons. This was confirmed by *in situ* hybridization on brain slices of P21 mice using a ^35^S-*Lat2* riboprobe combined with immunohistochemistry for NeuN in neurons and GFAP in astrocytes (Fig 1G, H). While neurons were clearly labeled, astrocytes were not. These results agree with the data from the transcriptomic database of Cahoy et al [27] that *Lat2* is expressed in neurons and not in astrocytes when primary brain cell cultures were analyzed, though in cultured astroglial cells *Lat2* expression becomes significant, explaining results from other authors [22].

### Thyroid hormone concentrations

**Figure 2:**
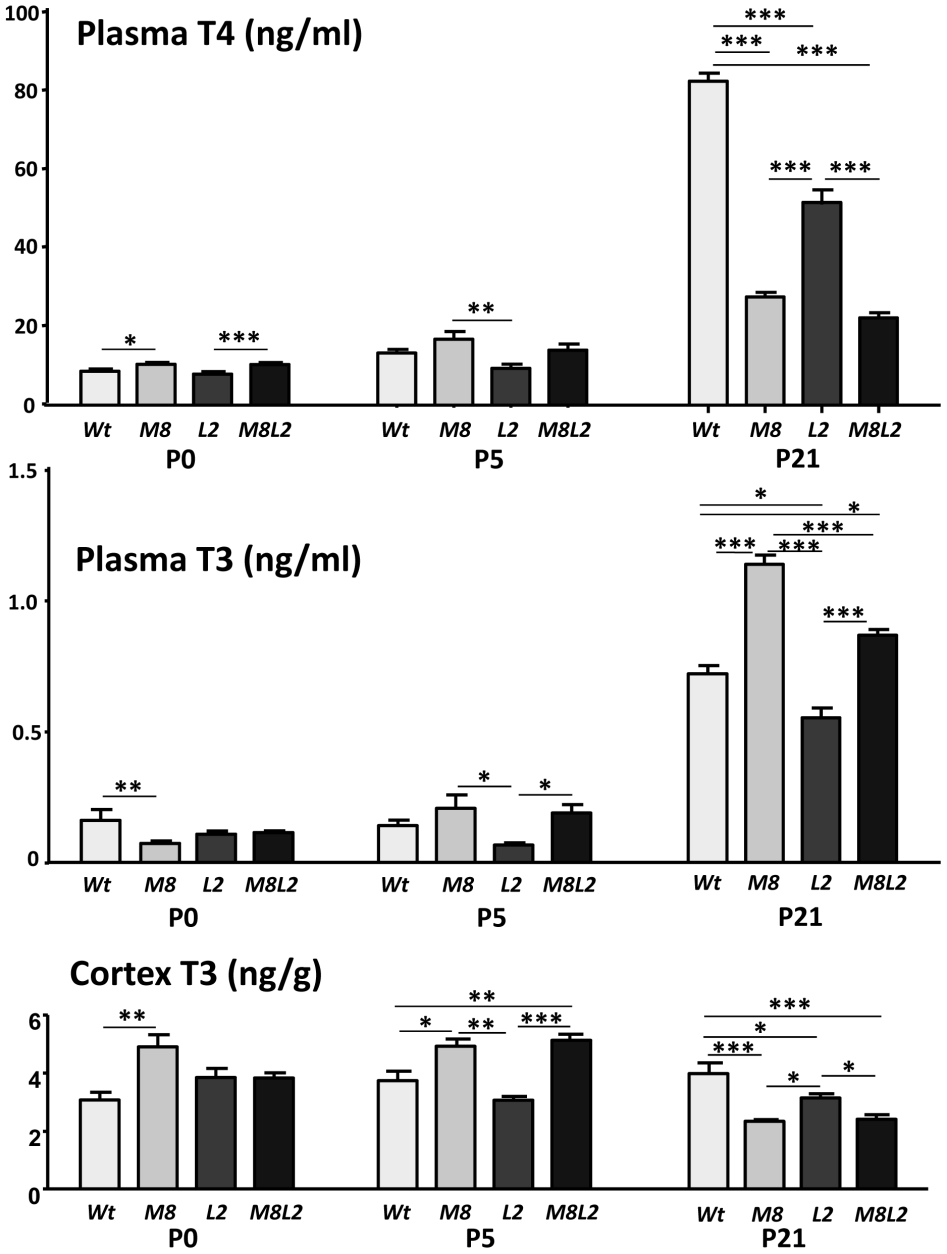
Plasma T4 and T3 and cortex T3 concentrations in mice of different genotypes and different ages, as indicated. *Wt*  =  wild type mice; M8 =  *Mct8KO*. L2 =  *Lat2KO*; M8L2 =  *Mct8Lat2KO*. Significance of differences was calculated by one way ANOVA, and the Tukey posthoc test. Only relevant significant comparisons are indicated. * P<0.05. ** P<0.01. *** P<0.001. P0, P5, and P21: postnatal days 0, 5, and 21, respectively.

The plasma T4 and T3 concentrations at P0, P5, and P21 are shown in figure 2. On P0 T4 was increased in the mice lacking Mct8, with significant differences between *M8* and *Wt* and between *M8L2* and *L2*. There was no difference between *Wt* and *L2*. By P5 the T4 concentration in the *M8* was still slightly elevated although the difference with the *Wt* was not significant. In the *L2* mice T4 showed a tendency to be lower than in the other groups, although the difference was significant only when compared to the *M8*. By P21 plasma T4 was 60% lower in the *M8* mice, as expected, and 35% in the *L2* mice compared to *Wt*. The *M8L2* mice showed similar T4 values as the *M8* indicating that the lack of Mct8 determined the T4 concentration in the double *KO*.

The plasma T3 concentration on P0 was reduced in the *M8* mice compared to the *Wt*. On P5 the mean T3 concentration was higher in the M8 than in the Wt, although the difference was not significant. T3 in the L2 was lower than in the *M8* or *L2M8* mice. By P21 plasma T3 showed the expected changes in the *M8* mice, with a 37% increase. Lat2 deficiency alone was associated with 25% decrease of T3. In the *M8L*2 mice T3 was lower than in the *M8* only mice, indicating that the absence of Lat2 moderated the effect of *Mct8* inactivation on the T3 concentration.

The bottom panel of Fig 2 shows the T3 concentrations in the cerebral cortex. On P0 there was a 40% increase of T3 in the *M8* mice, without changes in the *L2* or *M8L2* mice. On P5 cortex T3 was increased in the *M8* and the *M8L2* mice. On P21, T3 was decreased in the cortex of *M8* and *M8L2 mice*, in agreement with the known effect of *Mct8* inactivation at this age [11]. Inactivation of *Lat2* only also decreased T3 in the cortex at P21, correlating with the decreased serum T3 and T4.

### Effects of thyroid hormone transporter deficiency on cerebral cortex gene expression

**Figure 3:**
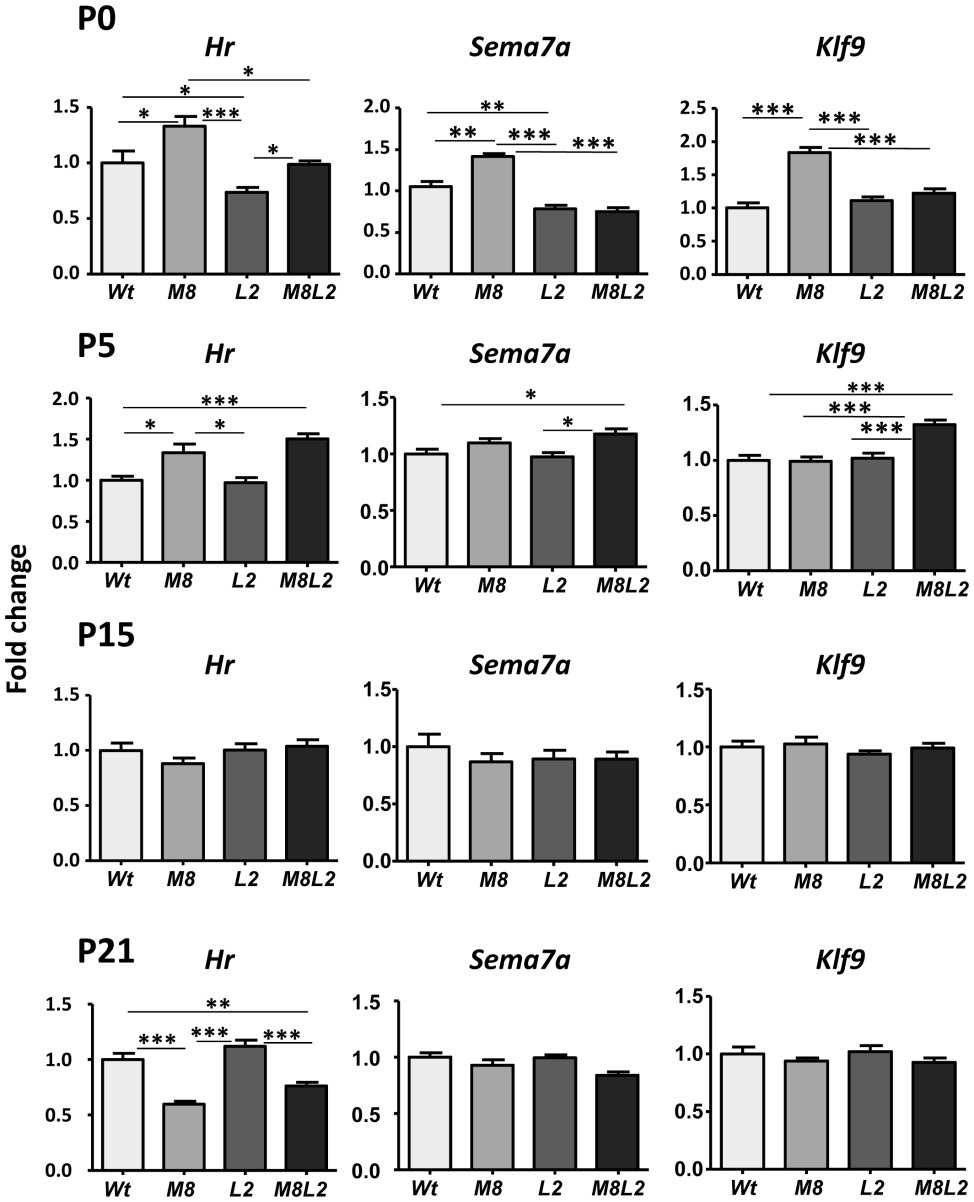
Gene expression (mean ± SE) in the cerebral cortex and liver of mice of different genotypes and ages as indicated. *Wt* =  wild type mice; M8  =  *Mct8KO*; L2 =  *Lat2KO*; M8L2 =  *Mct8Lat2KO*. Measurements were by qPCR, and the data expressed relative to 18S RNA. Significance of differences was calculated by one way ANOVA and the Tukey posthoc test. Only relevant significant comparisons are indicated. * P<0.05. ** P<0.01. *** P<0.001. *Hr*: Hairless mRNA. *Sema7a*: Semaphorin 7a mRNA. *Klf9*: Kruppel factor 9, or BTEB mRNA. P0, P5, P15, P21: postnatal days 0, 5, 15, or 21.

Fig 3 shows the effects of transporter inactivation on the expression of thyroid hormone-dependent genes in the cerebral cortex. RNA was prepared from the cerebral cortex of *Wt, M8, L2, and M8L2* mice at P0, P5, P15, and P21. Expression of three sensitive T3-responsive genes *Hr*, *Sema7a* and *Klf9* was measured by qPCR in all genotypes at all ages. In agreement with recent findings [23] at P0 *Hr* expression in the cortex was increased by 32% in the *M8* mice. We also found that *Hr* expression decreased in the *L2* mice by 27%. Remarkably, the double *M8L2* mice did not show the increased *Hr* expression over the *Wt* observed in the *M8* only mice, indicating that normal expression of *Lat2* was required for this effect. The two other genes, *Sema7a* and *Klf9* showed changes similar to *Hr*. *Sema7a* expression was increased by 35% in the *M8* mice, decreased by 25% in the *L2* mice, and again *Lat2* inactivation suppressed the effect of Mct8 deficiency. *Klf9* increased by 85% in the *M8*, and did not change in the L2 mice. As with *Hr* and *Sema7a*, the increase in *M8* was suppressed by *L2* inactivation in the *M8L2* mice. Other thyroid hormone-responsive genes, *Aldh1a1*, *Aldh1a3*, and *Slc1a3* showed no changes in any of the phenotypes (data not shown).

The increased *Hr* expression caused by *Mct8* inactivation at P0 was still observed at P5 with the important difference that at this age there was no suppressive effect of the additional *Lat2* inactivation. The *Sema7a* and *Klf9* responses were somewhat variable, with an increased expression in the *M8L2* mice. After a transition at P15, with similar expression of the three genes in all genotypes, a 40% reduction of *Hr* expression was observed at P21 in the M8, without changes in *Sema7a* and *Klf9*, in agreement with previous findings [14]. There were no effects of *Lat2* inactivation, and the combined Mct8Lat2 deficiency had the same effect as the Mct8 deficiency alone. For comparison, induction of hypothyroidism by administration of antithyroid drugs [13] caused a 38% decrease of *Hr* expression at P0 and 85% decrease at P21 (data not shown).

**Figure 4:**
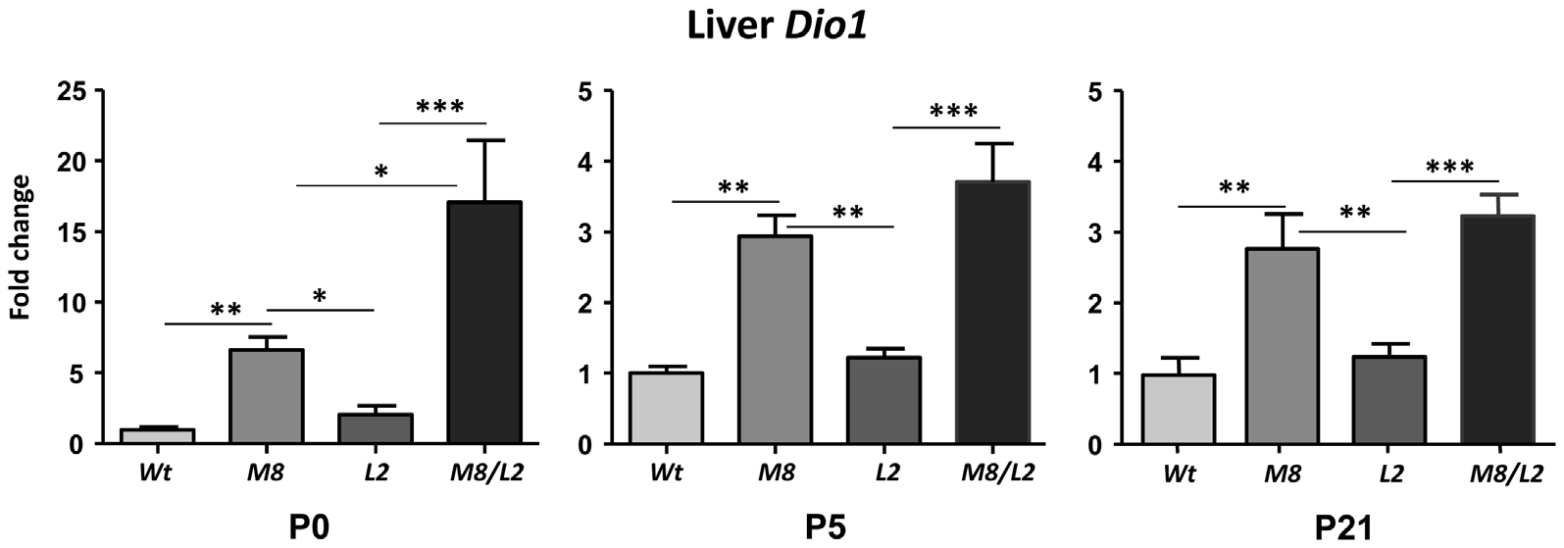
Type 1 deiodinase (*Dio1*) expression (mean ± SE) in the liver. *Wt* =  wild type mice; M8 =  *Mct8KO*; L2 =  *Lat2KO*; M8L2 =  *Mct8Lat2KO*. Measurements were by qPCR, and the data expressed relative to 18S RNA. Note the different scale for the P0 data with respect to P5 and P21. Significance of differences was calculated by one way ANOVA and the Tukey posthoc test. Only relevant significant comparisons are indicated. ** P<0.01. *** P<0.001. P5, P21: postnatal days 5 or 21.

Finally, we measured the expression of liver *Dio1,* a sensitive marker of peripheral thyroid status [10], [28] (Fig 4). At P0 *Dio1* mRNA abundance was very low, near the limit of detection by qPCR, and was more easily measured in the rest of the groups due to an increased expression over the *Wt* values. In the *M8* mice *Dio1* expression increased 7 fold at P0 and 3 fold at P5-P21. While the single *Lat2* inactivation did not modify *Dio1* expression, the combined *Lat2* and *Mct8* inactivation led to a synergistic 15 fold increase in the *M8L2* mice at P0. At P5 the *M8L2* mice still showed the highest mean value of all groups but the difference from *M8* was not significant.

## Discussion

The goal of the present work was to evaluate the contribution of Lat2, as a secondary thyroid hormone transporter, to thyroid hormone action in the brain. Our approach was to study the expression of thyroid hormone dependent genes in the cerebral cortex in a mouse deficient in Lat2. However, given the possible redundancy of different transporters we also analyzed the effect of Lat2 deficiency in the absence of Mct8. The reason for this approach is that, even if no effect of Lat2 deficiency only were observed, it is entirely possible that Lat2 cooperates with Mct8 and other transporters in thyroid hormone metabolism and action. In this context, the thyroid hormone transporter function of Lat2 [12], like that of other transporters such as Oatp1c1 [16], [29], might compensate for the Mct8 deficiency in the mouse brain. Lack of similar compensation in the human brain might explain the discordant neurological phenotypes of *Mct8KO* mice and MCT8 deficient patients.

A previous analysis of a different strain of *Lat2*KO mice found in adult mice a mild phenotype, with aminoaciduria, normal growth, and altered performance in the rotarod test, indicating light neurobehavioral alterations [30]. Mice had no obvious defects of thyroid hormone signaling, and had normal serum concentrations of thyroid hormones and TSH, normal cerebellar development, and normal expression of the T3 target genes *RC3* (*Nrgn*), *Hr*, and *Dio3* in the cerebellum and the cerebral cortex, and of *Dio1* in liver and kidney. In general these results agree with ours, and we did not find signs of delayed cerebellar development in the *Lat2*KO only mice as well as in the double *Mct8Lat2KO* mice (results not shown). We also found altered rotarod performance in the adult *Lat2KO* mice that was not worsened by concomitant Mct8 deficiency (data not shown). In our strain of mice, Lat2-deficiency had a mild effect on circulating thyroid hormone concentrations in juvenile animals, with decreased T4 and slightly decreased T3 at P21. These hormonal changes, however, did not result in tissue hypothyroidism, in liver or brain, with normal expression of *Dio1* and *Hr*, respectively. On the other hand the Mct8-deficient mice had the expected changes of circulating T4 and T3 [10], and the double *Mct8Lat2KO* mice mostly resemble the *Mct8KO* only mouse. Increased *Dio1* mRNA in the *Mct8KO* or *Mct8Lat2KO* at P21 paralleled the plasma T3 increase in these groups. Our findings, together with the previous findings by Braun et al [30] indicate that Lat2 has little contribution to thyroid hormone economy at least from the late postnatal period.

Despite this conclusion, Lat2 might be relevant to thyroid hormone transport in the brain during the perinatal period. As already reported [23] the newborn *Mct8KO* mice showed an unexpected cerebral cortex hyperthyroidism as reflected in the expression of the thyroid hormone-regulated genes *Hr*, *Sema7a*, and *Klf9*, which is not due to immaturity of the brain barriers [23], [31]. Lat2 is required for the hyperthyroid effect of Mct8 deficiency, at least at P0. At this age Lat2 deficiency blocks the increased gene expression and cortex T3 concentration observed in the absence of Mct8. However the specific pathway controlled by Lat2 is not apparent. As in the earlier study [23] we found here that plasma T4 was increased in the *Mct8KO* mice, and also in the *Mct8Lat2KO*. T3 was however decreased in the plasma at P0 supporting the view that cerebral hyperthyroidism is not due to increased uptake of T3 by the brain, and correlates better with the increased circulating T4. This suggests that the cortex hyperthyroidism is due to either increased local production of T3 from T4, or to retention of T3 in the cortex. The latter could indicate that the absence of Mct8 interferes with T3 efflux [23] and/or with its degradation by D3 in neurons [32] leading to T3 accumulation. The specific role of Lat2 is not evident but given its neuronal expression, it is reasonable to think that it has relevant role in T3 influx into the neurons during the perinatal period.

Liver *Dio1* was increased in the *M8* mice at P0 as already reported [23]. This change takes place in the face of a lower plasma T3 concentration than in the *Wt*, as shown in this work, and in the presence of an increased hepatic T4 content at E18 and P0 [23] consistent with a role of Mct8 in thyroid hormone efflux. Interestingly we found that the Lat2 and Mct8 deficiencies have a synergistic effect on *Dio1* expression at P0, reminiscent of what happens with the combined Mct8 and Mct10 deficiency in older animals [33]. This supports the view that during the perinatal period Lat2 has a role in thyroid hormone efflux at least in the liver.
